# Demonstration of efficient vertical and venereal transmission of dengue virus type-2 in a genetically diverse laboratory strain of *Aedes aegypti*

**DOI:** 10.1371/journal.pntd.0006754

**Published:** 2018-08-31

**Authors:** Irma Sánchez-Vargas, Laura C. Harrington, Jeffrey B. Doty, William C. Black, Ken E. Olson

**Affiliations:** 1 Arthropod-borne Infectious Diseases Laboratory, Department of Microbiology, Immunology and Pathology, Colorado State University, Ft. Collins, CO, United States of America; 2 Department of Entomology, Cornell University, Ithaca, NY, United States of America; 3 Centers for Disease Control and Prevention, Poxvirus and Rabies Branch, Atlanta, Georgia, United States of America; INDEPENDENT RESEARCHER, UNITED STATES

## Abstract

*Aedes aegypti* is the primary mosquito vector of dengue viruses (DENV; serotypes 1–4). Human-mosquito transmission cycles maintain DENV during epidemics but questions remain regarding how these viruses survive when human infections and vector abundance are minimal. *Aedes* mosquitoes can transmit DENV within the vector population through two alternate routes: vertical and venereal transmission (VT and VNT, respectively). We tested the efficiency of VT and VNT in a genetically diverse laboratory (GDLS) strain of *Ae*. *aegypti* orally infected with DENV2 (Jamaica 1409). We examined F1 larvae from infected females generated during the first and second gonotrophic cycles (E1 and E2) for viral envelope (E) antigen by amplifying virus in C6/36 cells and then performing an indirect immunofluorescence assay (IFA). RT-PCR/nested PCR analyses confirmed DENV2 RNA in samples positive by IFA. We observed VT of virus to larvae and adult male progeny and VNT of virus to uninfected virgin females after mating with males that had acquired virus by the VT route. We detected no DENV2 in 30 pools (20 larvae/pool) of F1 larvae following the first gonotrophic cycle, suggesting limited virus dissemination at 7 days post-infection. DENV2 was detected by IFA in 27 of 49 (55%) and 35 of 51 (68.6%) F1 larval pools (20 larvae/pool) from infected E2 females that received a second blood meal without virus at 10 or 21 days post-infection (E2-10d-F1 and E2-21-F1), respectively. The minimum filial infection rates by IFA for E2-10d-F1 and E2-21d-F1 mosquitoes were 1:36 and 1:29, respectively. The VNT rate from E2-10d-F1 males to virgin (uninfected) GDLS females was 31.6% (118 of 374) at 8 days post mating. Twenty one percent of VNT-infected females receiving a blood meal prior to mating had disseminated virus in their heads, suggesting a potential pathway for virus to re-enter the human-mosquito transmission cycle. This is the first report of VNT of DENV by male *Ae*. *aegypti* and the first demonstration of sexual transmission in *Aedes* by naturally infected males. Our results demonstrate the potential for VT and VNT of DENV in nature as mechanisms for virus maintenance during inter-epidemic periods.

## Author summary

*Aedes aegypti* mosquitoes are the primary vectors of dengue viruses (DENV) and other medically important arthropod-borne viruses (yellow fever, Zika, chikungunya). DENVs replicate in *Ae*. *aegypti* and humans in a transmission cycle that maintains the virus in nature. However, adverse conditions (i.e. dry seasons and low temperatures, vector populations, and host susceptibility) can suppress the transmission cycle. How does the virus survive in nature when the mosquito-human epidemic cycle subsides? Alternative DENV transmission routes can allow the virus to remain in the vector population. DENV can vertically transmit from mother to offspring and venereally transmit from infected male offspring to females during mating. In this report, we show that DENV2 efficiently pass from one generation to the next after the infected female mosquito acquires a second blood meal. Furthermore, infected male progeny arising from vertical transmission can then infect females during mating. Vertical transmission of DENV2, possibly in combination with venereal transmission, can potentially maintain DENV2 for sufficient time for the vector-human transmission cycle to resume.

## Introduction

Dengue viruses (DENV) include four serologically related but genetically distinct viruses, DENV1-4 (*Flavivirus*; *Flaviviridae*). DENV are arthropod-borne viruses (arboviruses) transmitted between susceptible humans by the bite of infected *Aedes spp*. DENV and their mosquito vectors are present throughout the tropics causing an estimated 390 million human infections per year [1]. DEN disease outbreaks in Florida and Hawaii have heightened awareness and concern of DENV transmission in the USA [2–4]. *Ae*. *aegypti* is the most important mosquito vector during large DENV outbreaks in tropical and subtropical urban regions [3]. DENV epidemics occur when mosquitoes and susceptible humans are sufficiently plentiful to maintain the vector-human transmission cycle. *Ae*. *aegypti* take multiple blood-meals during each gonotrophic cycle [5–8]. Ingestion of multiple uninfected blood feedings boost a previous incipient infection, enhancing virus dissemination to secondary tissues and transmission [9]. This effect was also observed for Zika virus (ZIKV) in *Aedes albopictus* [10]. Vertical transmission (VT) from one generation to the next and venereal transmission (VNT) during mosquito mating are mechanisms of DENV transmission within *Aedes* populations [11–13]. The potential impact of VT and VNT in sustaining DENV in the vector population remains unclear. Given the large public health consequences of DENV infections globally, a determination of the role VT and VNT plays in DENV maintenance in mosquito populations could lead to a more complete understanding of DENV persistence in nature and new insights into DENV transmission dynamics.

VT of arboviruses occurs by either transovarial transmission (TOT), in which the virus infects germinal tissues of the female (including oocytes) [14], and by trans-ovum transmission, which occurs at the time of fertilization or by virus contamination of the egg surface during oviposition [14, 15]. VT mechanisms are not mutually exclusive. In this report, we define VT rate (VTR) as the number of infected females in a population that produce at least one infected offspring. The filial infection rate (FIR) is the proportion of infected progeny produced from infected parents, given that VT has occurred. The minimum filial infection rate (MFIR) is the total number of positive mosquito pools divided by total mosquitoes and the effective VTR (eVTR) is the average number of infected progeny per infected female (VTR multiplied by the FIR) [4, 16, 17].

*Ae*. *aegypti* typically become infected when the female ingests a blood meal from a viremic human. *Ae*. *aegypti* females lay their eggs within days of acquiring a blood meal. *Ae*. *aegypti* embryos can become dormant at the end of embryogenesis and remain viable inside the egg for up to six months [18]. When conditions become favorable for larval development, dormancy is interrupted and the eggs hatch to larvae [19, 20]. Flaviviruses that persist in eggs can be transmitted transstadially from larvae to pupae to adults [13]. Infected females potentially generate hundreds of eggs in their lifetime providing opportunities for VT and VNT within the vector population.

In early studies, Rosen detected minimal VT of DENV among *Ae*. *aegypti* [15, 21] but more recent reports have provided consistent evidence of VT in field-collected *Ae*. *aegypti* [22–25], although significant variations in the frequency of VT have been noted among strains of *Ae*. *aegypti* infected with different DENV serotypes and genotypes [12]. DENV have been detected in field-collected larvae [22] and field caught adult male mosquitoes [26–28]. The detection of DENV infected male mosquitoes suggests that males could play a role in the maintenance of DENV in nature [29]. Few studies have addressed the role of male mosquitoes in DENV transmission dynamics and only one study focused on VNT of DENV in *Ae*. *albopictus* [11] by determining VNT by males that were artificially injected with virus. In this context, we tested VT and VNT of DENV2 in a genetically diverse laboratory strain (GDLS) of *Ae*. *aegypti* derived from mosquito populations collected in Chiapas, Mexico [30]. Initially, we investigated whether VT in GDLS was more efficient in the first or second gonotrophic cycle (E1 and E2). We clearly observed that orally infected GDLS mosquitoes vertically transmitted DENV2 to their F1 progeny during the second gonotrophic cycle (E2) and VT increased if a second blood meal was given 10 or 21 days post infection. Significantly, the MFIR determined by either RT-semi-nested-PCR (RT-N-PCR) or IFA was 8–10 fold higher than previously reported for flaviviruses [17, 31]. We detected DENV2 in ovarian tissues and oocytes of infected females suggesting VT can occur by TOT. The present study also demonstrated that males acquiring DENV2 by the VT route were capable of transmitting the virus to un-infected virgin GDLS females during mating. Our results suggest that VT and VNT of DENV in mosquito populations are potential mechanisms for virus maintenance during inter-epidemic periods.

## Materials and methods

### Virus and cell cultures

LLC-MK2 and C6/36 (*Ae*. *albopictus*) cells were cultured in modified Eagle’s medium (MEM) supplemented with 8% fetal bovine serum, L-glutamine, non-essential amino acids and penicillin/streptomycin and maintained at 37°C, 5% CO_2_ and 28°C, 5%CO_2_, respectively. DENV2-JAM1409 strain [32] was used to infect fresh cultures of C6/36 cells to prepare infectious blood meals. DENV2-JAM1409 belongs to the American-Asian genotype [33, 34] and was originally obtained from the Centers for Disease Control and Prevention (CDC- Fort Collins, CO, USA). The virus has been routinely passaged (> 25 times) in C6/36 cell culture. Briefly, monolayers of C6/36 cells were exposed to DENV2 at a multiplicity of infection (MOI) of 0.01 and incubated at 28°C; 6 days later medium was replaced and infected cells and medium were harvested at 12–14 days to prepare the blood meal [35].

### Mosquito rearing and DENV2 infection

The *Ae*. *aegypti* GDLS strain was derived from a mixture of equal numbers of 10 geographically distinct collections made in 2008 from Chiapas, Mexico [30] and eggs were hatched and reared to adults (28°C/75-80% relative humidity (RH); photocycle of 16:8 L:D). To infect mosquitoes, groups of 100–150 one-week-old adult females were placed in 2.5 L cartons, deprived of sugar and water overnight and allowed to feed on artificial blood meals consisting of virus-infected C6/36 cell suspension (60% vol/vol), 40% (vol/vol) defibrinated sheep blood (Colorado Serum Co., Boulder, CO) and 1 mM ATP [36]. Mosquitoes fed for 60 min with blood meals contained viral titers of 2.7 ± 2 × 10^6^ PFU/ml. We selected engorged females, incubated them for up to 17 days (28°C and 75–80% RH) and then analyzed to determine the infection rate. Virus in the blood meal was quantified by plaque titration on LLC-MK2 cells as previously described [37].

### C6/36 cell infection and IFA

Tissue from whole 4^th^ instar larvae, adult heads, or the male or female reproductive tracts were homogenized, filtered (Acrodisc Syringe filters with 0.22 μm HT Tuffryn membrane) and filtrates used to infect 12-well plates containing C6/36 cell monolayers on round cover slips. A positive control of homogenate from *Ae*. *aegypti* intrathoracically injected with DENV2 and a negative control (uninfected mosquito) were included in each assay. The cells were maintained at 28°C, 5% CO_2_ for 7 days and DENV2 E antigen detected using an IFA with 3H5 mAb as previously described [38–40].

### RT-N-PCR for detection of viral RNA

Trizol (Invitrogen, Carlsbad, California) was used for RNA extraction from mosquito homogenates following the manufacturer’s recommendations. Detection of viral RNA was performed using a semi-nested PCR described by Gunther et al. [41] with the following modifications: Primers for the NS3 gene of DENV2 [41, 42] were used to generate a 362 bp amplicon. The cDNA was amplified in a second step (nested PCR), using 3 μl of DNA from the RT-PCR reaction in the presence of forward primer (5’AATTGTCGACAGAAAAGGAAA 3’) and reverse primer (5’ GGCTGGGGTTTGGTATC 3’). The reaction contained 2X PCR master mix (Promega M750B), followed by 30 cycles of 94°C for 30 sec, 55°C for 1 min, 72°C for 1 min, with an additional extension step of 72°C for 10 min and held at 4°C [43].

### Vertical transmission assays

Female GDLS mosquitoes were fed a blood meal containing >10^6^ pfu/mL of DENV2 and held in a cage containing an oviposition cup. We analyzed E1 eggs (E1-F1) for VTR. After the first oviposition cycle, females were offered a second blood meal with no virus (BMnV) at either 7, 10 or 21 days pi (E2-7d, E2-10d and E2-21d respectively) to support E2 egg production (E2-F1). Blood-engorged females were maintained at 28°C and 80%RH in the insectary as previously described. Non-blood fed females were removed after the second blood meal and stored at -80°C. Virus titrations determined DENV2 infection rates from the initial infectious blood meal. Eggs from each gonotrophic cycle were collected for VT analyses.

To evaluate TOT, E1 and E2 eggs from DENV2-infected females were surface-sterilized by soaking in 0.05% sodium hypochlorite for 5–10 min, then for 1 min (3 times) in 70% ethyl alcohol, and finally washed with distilled water three times [17] before hatching. Fourth instar larval progeny (F1) were collected in pools of 20 and stored at -80°C. Each pool of larvae was homogenized and filtered prior to infecting C6/36 cell monolayers grown on coverslips in 12- well plates. Cells were analyzed by IFA for DENV2 E antigen as previously described. Total RNA was extracted from the pellet to conduct RT-N-PCR for confirmatory analysis of viral RNA.

### Venereal transmission assays

GDLS females were offered a blood meal containing ~10^6^ pfu/mL of DENV2 JAM1409 and a second BMnV at 10 dpi. GDLS females engorged with a second BMnV were transferred and held individually in 50-ml centrifuge tubes lined with a dry strip of paper towel. Distilled water was added one day after blood feeding to support egg production. E2 eggs (E2-10d-F1) from each female were stored in individual plastic bags. Each female was stored at -80°C in a numbered vial prior to testing for infection status. *Ae*. *aegypti* E2 eggs from each DENV2 positive female were hatched to develop to F1 adults. Males were separated at the pupal stage and adult females stored at -80°C. FIR was determined later. ***Mating*.** Individual E2-10d-F1 male progeny (5–6 days post-eclosion) transferred into small cardboard cartons were separated into two groups (Group I and II). Two sugar-fed uninfected, virgin females were added to Group I cartons prior to mating. Two uninfected, virgin females that had received a BMnV three days previously were added to Group II cartons prior to mating. Following a 48-h mating period, males were removed from the cartons and their reproductive tracts (testes, accessory glands and seminal vesicle) obtained by severing the last two abdominal segments and stored at -80°C prior to determining DENV2 infection status. The mated females were incubated at 28°C in Group I and II cartons until 8 days post mating. Thereafter, female reproductive tracts (ovaries and spermathecae) were dissected for analysis. Additionally, female mosquito carcasses (without reproductive tracts) were stored at -80°C. Male and female reproductive tissues were homogenized, filtered and filtrates used to infect C6/36 cells to detect DENV2 following the same procedures used for virus detection in larvae described above. We then tested by RT-N-PCR the heads of females (already shown to be virus positive by IFA in their reproductive tract) to observe DENV2 dissemination.

### Data analysis

The average proportion of infected female reproductive tracts, female carcasses, and male reproductive tracts were calculated by averaging the number infected divided by the total tested. The proportion of infected males that transferred virus to females was determined for each pairing. All data were analyzed with GraphPad prism software (version 5.0, La Jolla, CA, USA) was used to test for significant differences (*P* < 0.05) in TOT and FIR (by Fisher’s exact test) and to analyze the correlation between female parent titer and proportion on infected progeny (Pearson correlation). Analysis of variance (ANOVA) was used to determine the statistical significance of acquiring a second blood meal on virus replication.

## Results

### Infection and dissemination of DENV2 in GDLS mosquitoes

**Fig 1:**
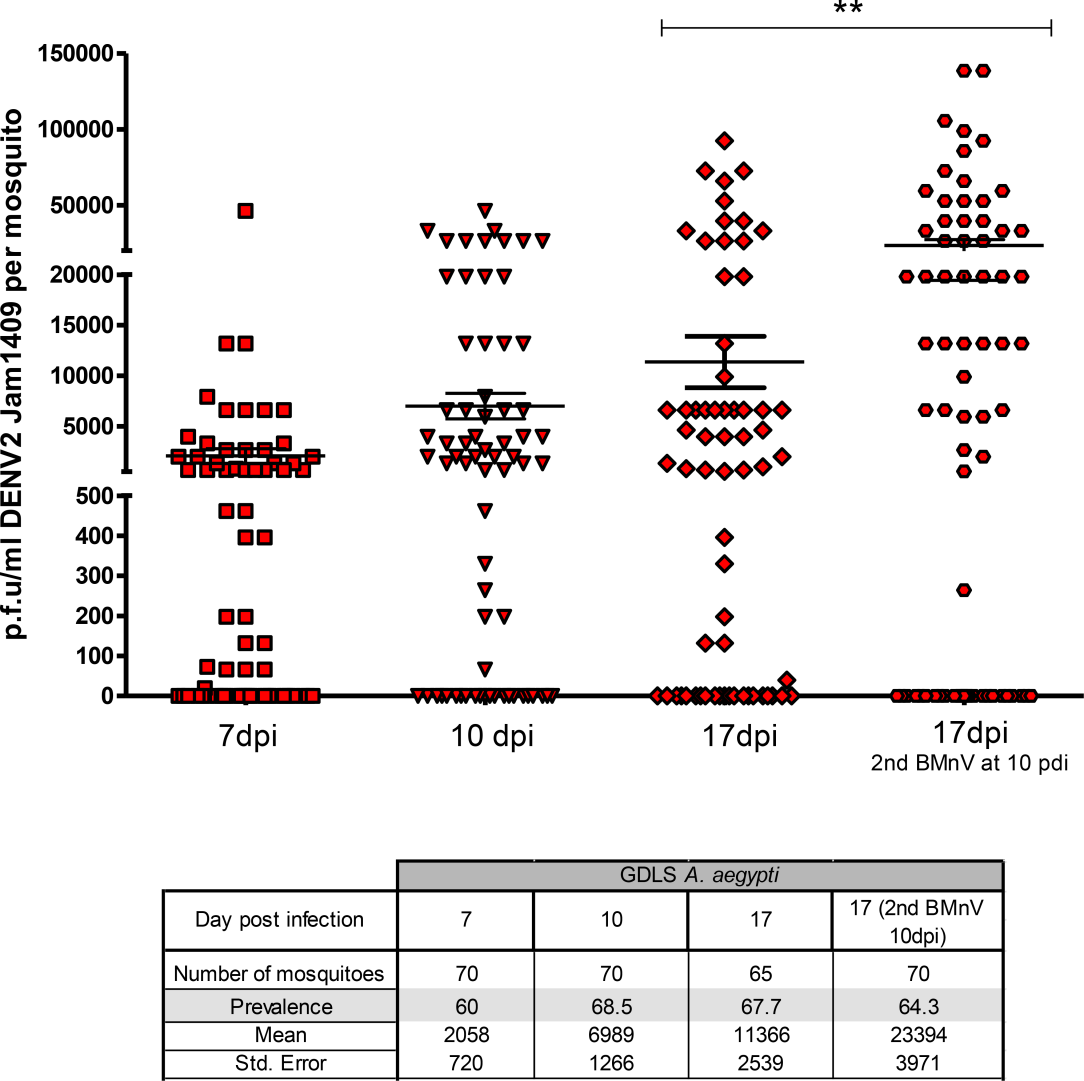
DENV2 JAM1409 infection of GDLS *Ae*. *aegypti*. GDLS mosquitoes were blood fed with DENV2 Jamaica1409 and virus titers of individual mosquitoes were determined at different day’s post-infection (dpi) by plaque assay. The number of mosquitoes assayed from each group, percentage of infection, means titers and blood meal titers are shown. Bars indicate mean values of titers ±SEM, ** P<0.0002. BMnV = blood meal no virus.

Since vector competence among *Ae*. *aegypti* populations varies according to the infecting DENV serotype and genotype and the genotype of mosquitoes, we initially evaluated the competence of *Ae*. *aegypti* (GDLS) strain for DENV2-JAM1409. Vector competence was analyzed by offering mosquitoes a blood meal containing 1×10^6^ to 4 ×10^6^ PFU/mL DENV2-JAM1409. We selected and assayed engorged mosquitoes for DENV2 at 7, 10, and 17 days post-infection (dpi) by plaque assay. The prevalence of DENV2 in *Ae*. *aegypti* (GDLS) was calculated as the proportion of mosquitoes infected among total mosquitoes receiving an infectious blood meal. The prevalence of DENV2 in GDLS females was approximately 65% (viral titers: 2 +/- 0.72 ×10^3^, 7 +/- 1.3 ×10^3^ and 1.1 +/- 0.25 ×10^4^ PFU/mL (per mosquito) at 7, 10 and 17 dpi, respectively (Fig 1). Midgut infection rates (MIR) and disseminated infection rates (DIR) were determined by detection of DENV-2 E antigen in midgut and head tissues (sampled pairwise) at 14 and 17 dpi. MIRs were 78.3% (18/23), 76.7% (23/30) and 75.7% (25/33) at 7, 14 and 17 dpi, respectively and DIRs were 63.3% (19/30) at 14 dpi and 81.8% (27/33) at 17dpi. DIRs were not calculated for the 7-day time point due to our previous studies that showed minimal dissemination at that time [44]. The MIRs and DIRs indicated that GDLS mosquitoes were highly susceptible and competent for DENV2-JAM1409.

*Per os* infection of *Ae*. *aegypti* with DENV2-JAM1409 has been described spatially and temporally in several *Ae*. *aegypti* populations [7, 45]. However, in those studies mosquitoes used a single infectious blood meal. In nature, *Ae*. *aegypti* females feed frequently and may ingest 2 to 3 blood meals during a single gonotrophic cycle [8]. Therefore, we determined DENV2 titers in mosquitoes receiving a second BMnV. The DENV2 titers at 17 dpi were significantly higher in GDLS females receiving a second BMnV at 10 dpi than GDLS females receiving a single, infectious blood meal (T test, *P*<0.0133. r = 0.0421) (Fig 1) however, no difference was observed in either prevalence or DIRs in the two groups. Several studies have shown that offspring produced during the second or later gonotrophic cycles display higher VT [13, 46, 47]. These observations may be linked to increased dissemination to the ovaries over time and it has been postulated that expanded parous ovaries, with stretched and squeezed follicular epithelial cells facilitate higher viral infection rates of oocytes, contributing to increased VT in subsequent cycles [48, 49].

**Fig 2:**
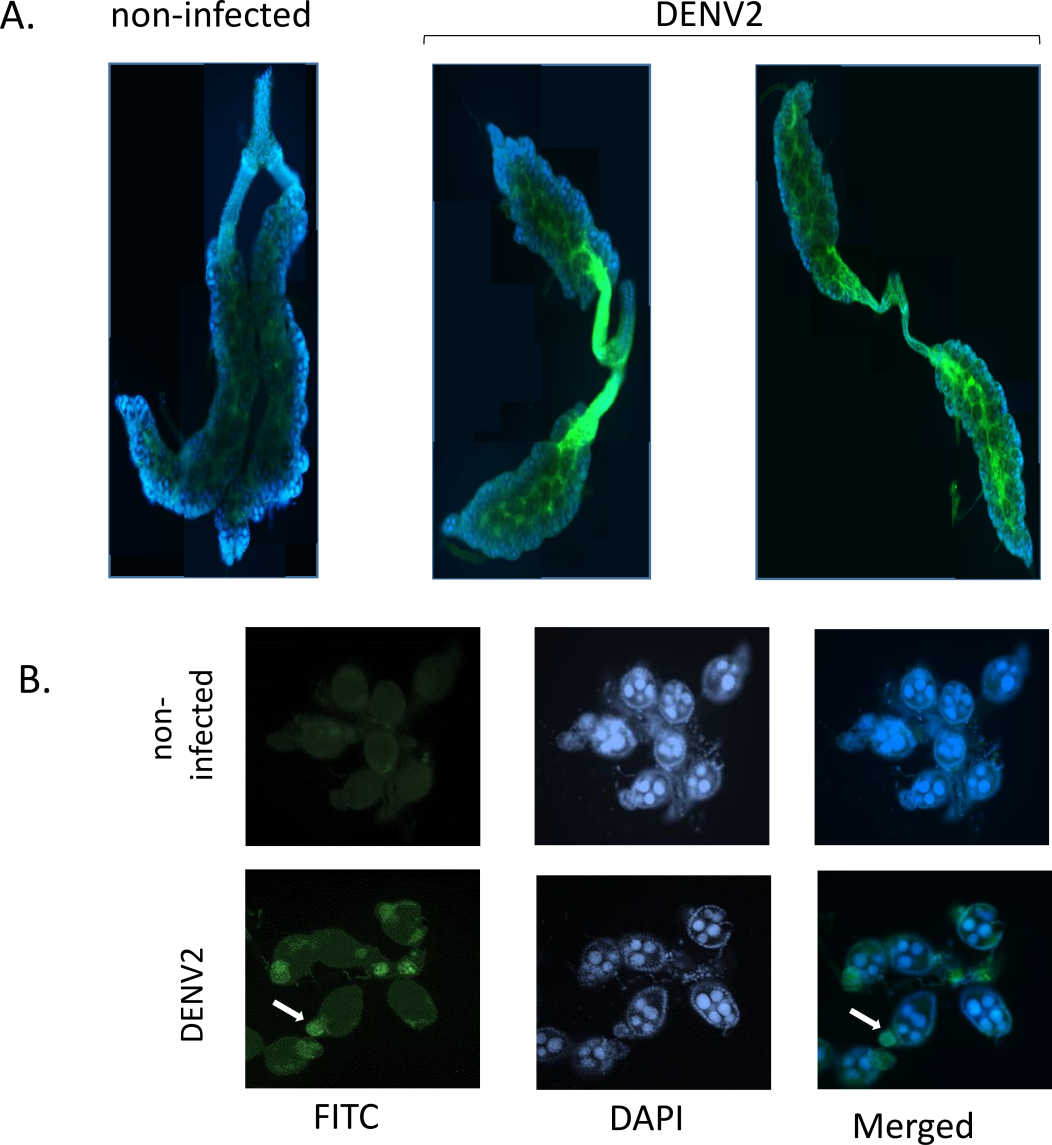
IFA of ovaries and oocytes dissected from females infected with DENV2 after second gonotrophic cycle. **A**. Microscopy images of ovaries of Groups I and II from control (left) and DENV2 infected females (right), showing infected ovaries and oviducts. **B**. Microscopy images of single oocytes from Group II females infected with DENV2. Arrows denote IFA positive germarium and follicles. DAPI signal (blue) and DENV2 antigen (FITC/green).

With this in mind, we tested the presence of the virus in the ovaries in DENV2 infected female mosquitoes. After the first oviposition cycle, females were separated into two groups. Group I females did not receive a second BMnV, Group II females were offered a second BMnV at 10 dpi and allowed to oviposit autogenously produced eggs. At 17 dpi, ovaries from both groups were dissected in PBS and fixed in 4% paraformaldehyde (Electron Microscopy, Hatfield, PA) for at least 4 h. DENV2 E antigen was detected using IFA with 3H5 mAb as previously described [38–40]. DENV2 antigen was detected in ovaries and oviducts in both groups at 17 dpi (Fig 2A). IFA staining was less intense in ovaries of Group I females than in Group II females. We observed infection of a small number of oocytes in ovaries of Group II females (Fig 2B). Oocyte infections demonstrated the potential for TOT, a VT pathway more efficient than trans-ovum transmission [50]. No infection was detected in ovaries from females of each group at 10 dpi suggesting VT by TOT is dependent on time and the number of blood meals ingested.

### Vertical transmission of DENV-2 as detected in 4^th^ instar larvae (F1 progeny)

**Fig 3:**
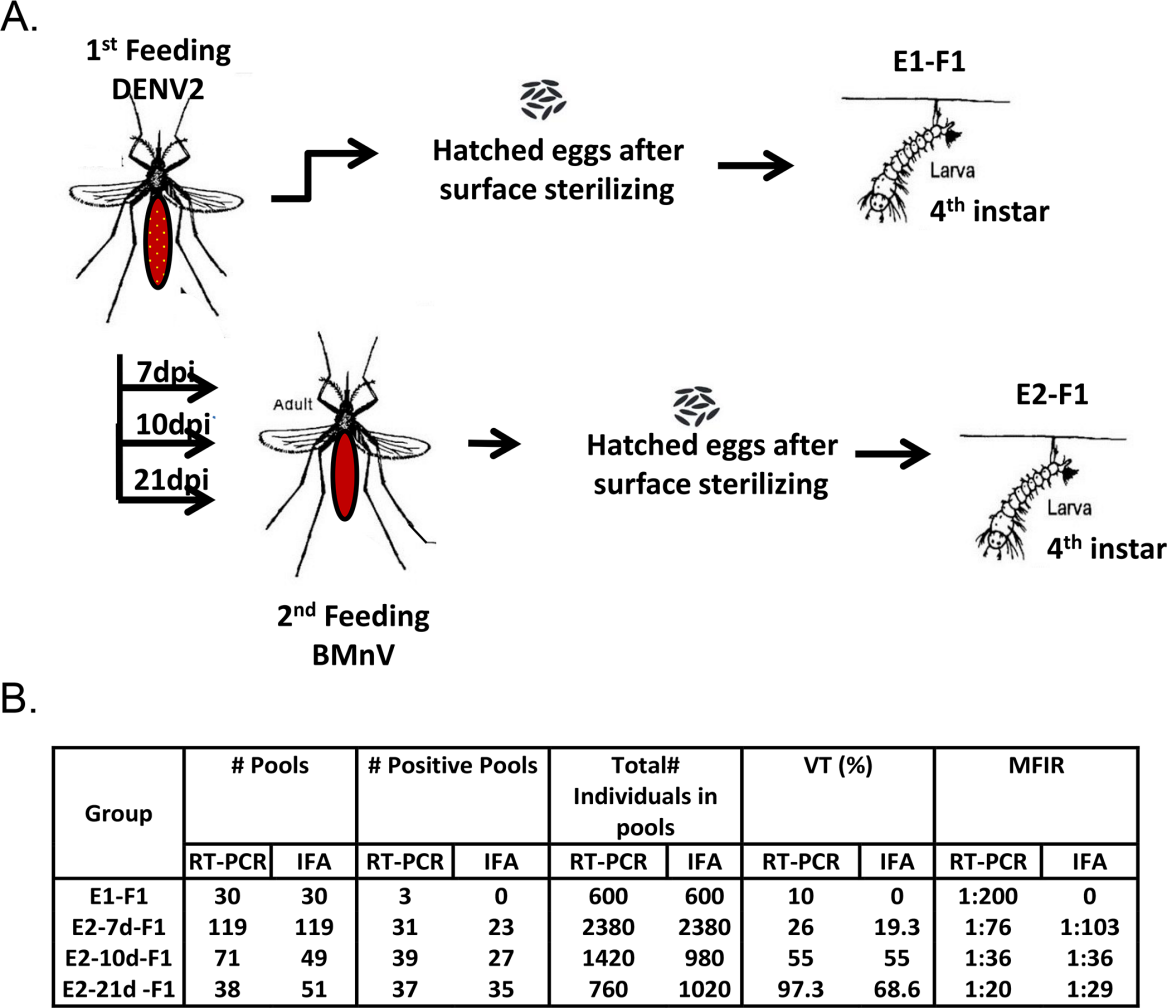
Vertical transmission of DENV2 to 4^th^ instar larvae. **A**. Schematic of steps followed to obtain F1 progeny 4^th^ instar larvae samples from the first (E1) and second (E2) gonotrophic cycle of GDLS mosquitoes infected orally with DENV2. After exposure to a blood meal containing virus, female mosquitoes were divided into four groups. Each group was offered no further blood meals (E1) a BMnV 7, 10 and 21 days later (E2-7d, E2-10d and E2-21d respectively). Eggs were collected for each group. After hatching, all larva pools were screened for the presence of DENV2. **B**. RT-N-PCR and IFA of infected C6/36 cells determined DENV2 minimum filial infection (MFIR) and vertical transmission rates (VTR).

We tested whether VT in GDLS was more efficient in the first or second gonotrophic cycle and if VT efficiency depended on the interplay between gonotrophic cycle and viral infection dynamics. Following oviposition, we collected eggs from each female. We then determined the infection status of all mothers but used only the eggs of virus positive females. The progeny were designated E1-F1 (Fig 3A). Infected GDLS females were given a second BMnV at either 7, 10 or 21 dpi to support E2 egg production and generate E2-F1 offspring. Eggs from the second cycle were collected and designated E2-7d-F1, E2-10d-F1 and E2-21d-F1 depending on the timing of the BMnV (Fig 3A). Since VTR mean values were not significantly different among three replicates for each designated collection, the data were analyzed as a single experiment.

DENV2 was not detected by IFA in 30 pools of larvae analyzed from the E1 progeny (infection rate = 0; 95% CI = 0.0–6.01) (Fig 3B). IFA readily detected VT of DENV2 in GDLS E2 progeny. For E2-7d mosquitoes, 23 of 119 (19.3%) pools were positive for DENV2. VTR increased when infected females received a second BMnV at 10 or 21 dpi with 55% (27 of 49) of larvae pools positive for E2-10d and 68.6% (35 of 51) positive for E2-21d. (Fig 3B). However, there was no significant difference in DENV2 positivity between pools of E2-10d and E2-21d (*P* = 0.22) larvae. A significant difference was observed between VT of E2-7d and E2-10d and between E2-7d and E2-21d (*P*<0.0001). The VTR and MFIR from larvae pools detected by RT-N-PCR were similar to results obtained by IFA (Fig 3B). The sequence of the amplicon confirmed that the DENV2 strain detected in E2-F1 larvae was the same as the one used to infect the parental female.

### Venereal transmission of DENV2

**Fig 4:**
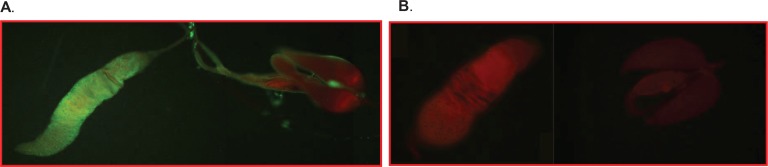
DENV2 infection in the reproductive tract of male mosquito progeny (E2-F1) of infected GDLS female. DENV2 E antigen was detected in testis and peripheral tissue of accessory glands by IFA using DENV2 specific 3H5 monoclonal antibody. **A**. Reproductive tract of male mosquito progeny (E2-10d-F1) from DENV2 infected GDLS female. **B.** Reproductive tract of male mosquito progeny from GDLS non-infected female.

Since we observed that GDLS mosquitoes infected with DENV2 vertically transmitted virus to larvae, we determined if F1 adult males were infected and if infected could, in turn, venereally transmit virus to virgin females. We tested virgin F1 male progeny arising from infected mothers to quantify FIR, VTR and VNT. GDLS females were given a blood meal containing >10^6^ pfu/mL of DENV2 JAM1409 and a 2^nd^ BMnV at 10 dpi. Mean DENV2 titers for infected mothers were 2.3 ± 6.5 ×10^2^ PFU/ml in replicate1 (R1), 9 ± 4.9 × 10^2^ PFU/ml in replicate 2 (R2) and 8.3 ± 1.3 × 10^3^ PFU/ml in replicate 3 (R3). E2 eggs from individual females positive for DENV2 were hatched to rear F1 adults. One 6-day-old male progeny (E2-10d-F1) mated with two uninfected, unfed, virgin females or virgin females that received a BMnV 3 days prior to mating. After mating, the male reproductive tracts (testes, seminal vesicle and accessory glands) from E2-10d-F1 were screened by IFA for DENV2 E antigen. We detected viral antigen in the testes and peripheral tissue of the accessory glands (Table 1, data R1; Fig 4). Dissected male reproductive tract homogenates were used to infect C6/36 cells. DENV2 E antigen was detected by IFA in cultured cells (Table 1, R2 and R3). The FIRs in F1 (E2-10d-F1) were 30, 27 and 30.5% for R1, R2 and R3; VTR was 48%, 57% and 52.4% and eVTR were 14.4%, 15.4% and 15.6% for R1, R2 and R3, respectively (Table 1). No statistically significant difference was observed in VTR between replicates (Fisher’s exact test, *P* = 0.144).

**Table 1:** Vertical and filial transmission rates in adult progeny (E2-10d-F1) from DENV2 infected females.

Replicate (R)	N (parent females)	FIR (%) *	VTR (%)	eVTR (%)
1	27	30	48	14.4
2	25	27	57	15.4
3	52	30.5	52.4	15.6

*based on F1 male progeny tested

**Fig 5:**
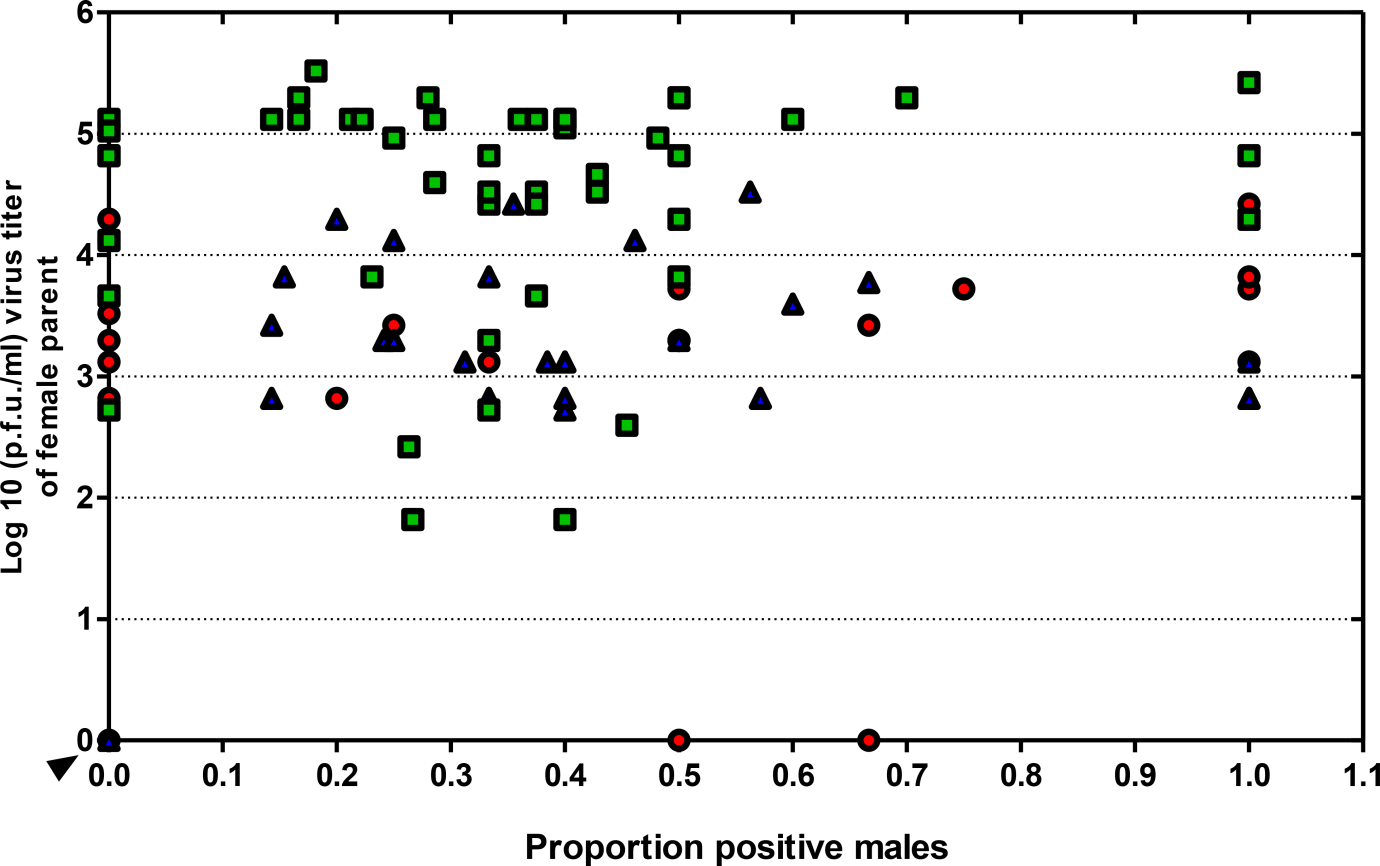
FIR in male progeny showing lack of correlation between DENV2 titer in the female parent and proportion of infected male progeny. DENV2 titers for each infected parental GDLS female within each replicate were measured separately to determine the MFIR in male progeny. Female mosquitoes were orally infected with DENV2 and received a second BMnV at 10 dpi. E2 eggs were collected from each female placed in numbered vials. Only E2 eggs from females positive for DENV2 were hatched to rear to F1 adults to determine MFIR. No significant correlation between female parent titer and proportion progeny infected was detected (Pearson correlation, r = 0.049, P = 0.804; r = 0.267, P = 0.176; r = 0.013, P = 0.933, respectively). Non-infected female mosquitoes with a non-infected male progeny (negative control) were clustered at the origin (arrow); 1^st^ replicate (red circle); 2^nd^ replicate (dark blue triangle); 3^rd^ replicate (green square).

No significant correlation was detected between DENV2 titer from the female parent and the proportion of progeny that were positive for virus among the replicates (Pearson correlation, r = 0.049, *P* = 0.804; r = 0.267, *P* = 0.176; r = 0.013, *P* = 0.933, for R1, R2 and R3 respectively) (Fig 5). DENV2 was not detected in E2-10d progeny from uninfected females. These results confirm VT to male progeny and suggest the potential for VNT of DENV2 from males to females during mating.

**Fig 6:**
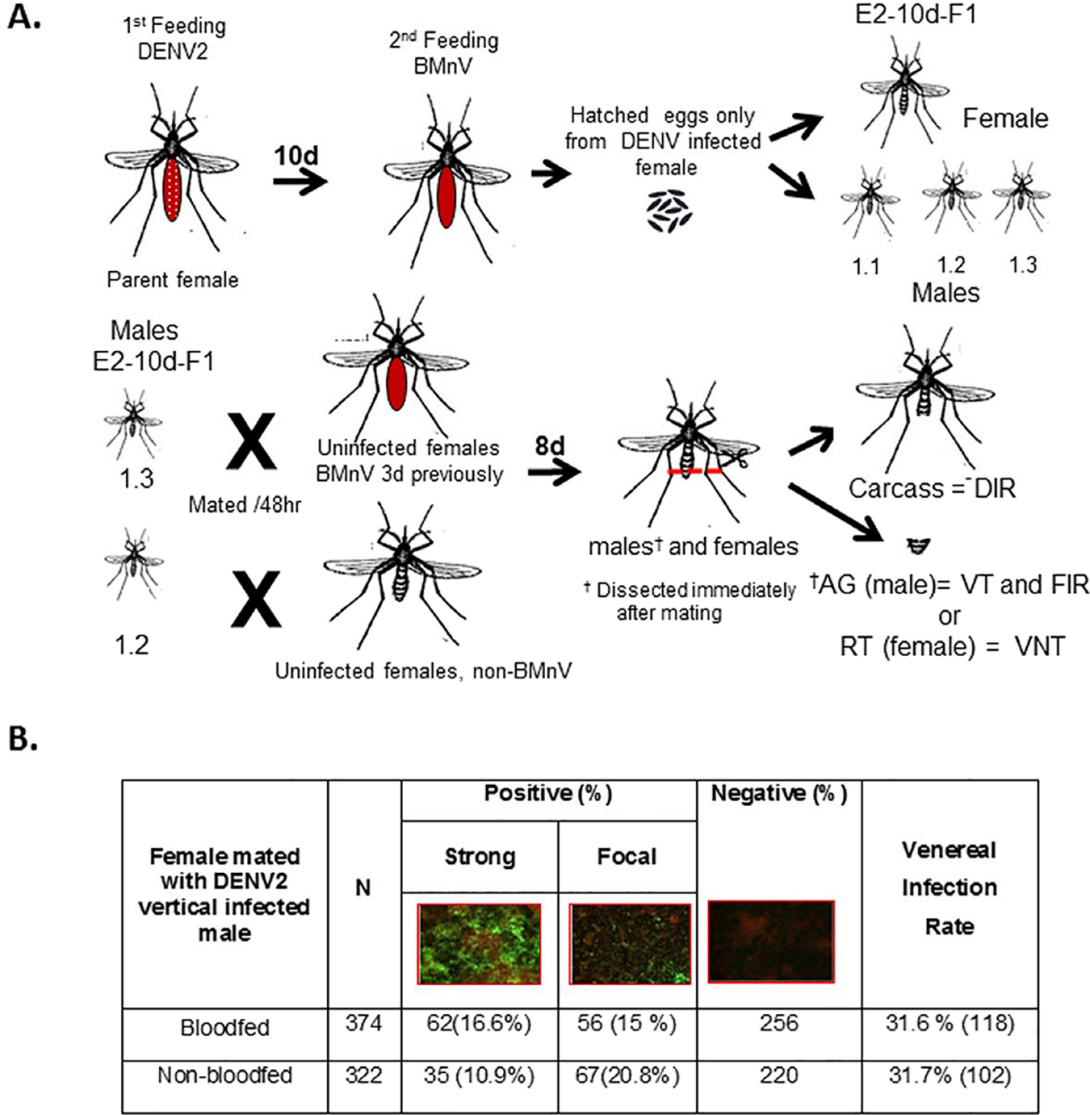
Venereal infection rates of GDLS females mated to males vertically infected with DENV2. **A**. Schematic of steps used in VNT assay. Male progeny (E2-10d-F1; designated 1.1,1.2, etc.) of DENV2 orally infected females were allowed to mate either with virgin non-infected females fed with non-infectious blood meal (3 days before) or non-blood fed at a ratio of 1:2. After mating, IFA determined infection status of reproductive tissues of males. Live females were maintained until 8 dpi at 28°C. Thereafter, infection rates were determined in female reproductive tracts by IFA following virus amplification in C6/36 cells. AG = male accessory gland; RT = female reproductive tract. **B**. Infection rates of non-infected females (fed with non-infectious blood meal or non-blood fed) mated with males vertically infected with DENV2.

We tested whether enhancement of VNT of DENV2 occurred if an uninfected female had a blood meal prior to mating and used the method outlined in Fig 6A. Other studies have observed that male *Ae*. *albopictus* experimentally infected with DENV 1, 2, 3, or 4 sexually transmitted virus to females [11] and VNT was enhanced if the females had taken a blood meal 2 to 7 days prior to mating [11, 51]. It also has been reported that uninfected female mosquitoes that were given blood meals 3–4 days prior to mating were more likely to develop systemic infections after mating with infected males than females that had never received a blood meal [51]. VT infected male mosquitoes (GDLS) were mated to either unfed, non-infected, virgin GDLS females or GDLS females that had received a BMnV three days prior to mating. After mating, the males were separated and infection rates of their reproductive tracts determined 8 days later by IFA. The infection rate of the males was 28.7% (140/488). We mated all F1 males with females but only the females that mated with DENV2 positive males were used to determine VNT rate. Female reproductive tracts were collected and infection rates determined by IFA and RT-N-PCR. The VNT rate was 31.6% (118 of 374) in blood fed females and 31.7% (102 of 322) in non-blood fed females. Data were analyzed as a single experiment since VNT rates among three replicates were not significantly different (blood fed, *P* = 0.16; non-blood fed *P* = 0.06; Fig 6B). VNT to blood fed and non-blood fed GDLS females were not significantly different (χ2< 0.001, df = 1, *P* = 0.97 Fisher’s exact test *P* = 1.00).

Additional VNT data were obtained using uninfected virgin males that had been intrathoracically inoculated with DENV2-JAM1409 (100 pfu) 14 days prior to mating. The infection rates in the reproductive tract of males were determined (RT-N-PCR and IFA) before mating by randomly sampling 20 mosquitoes at 12 dpi. DENV2 E antigen was detected in the testes and peripheral tissue of the accessory glands in 100% of the injected mosquitoes (Fig 4). These results were similar to those reported in ultrastructural studies of intrathoracically infected male mosquitoes in which DENV2 was detected in the testes, seminal vesicle and accessory glands [52]. However, no virus was found in the germ cells (spermatogonia, spermatocytes, spermatid, spermatozoa). The mechanism of sexual transmission of DENV2 from infected male to female mosquitoes remains unclear. Once DENV2 was confirmed within reproductive tract of males, the remaining mosquitoes were allowed to mate following the same protocol previously described. Female reproductive tracts were collected 8 d after mating and infection rates were determined with RT-N-PCR and IFA. Forty three percent (12/28) of females were positive for DENV2 by IFA. VNT rates for females receiving a BMnV prior to mating and those receiving no BMnV were not significantly different (*P* = 0.20). The VNT rate was slightly higher using the inoculated males although not significantly different from VNT rates using males that had acquired their infection by the VT route and regardless of the mates BMnV feeding status (bloodfed, P = 0.216; non-bloodfed P = 0.154). The data demonstrate that DENV2 vertically or artificially infected males were capable of transmitting virus efficiently to females during mating.

To determine if virus acquired by VNT could disseminate, we tested heads of BMnV and non-BMnV females by RT-N-PCR. We did not test experimental transmission of the virus in saliva, but we did detect the presence of virus in the head of female mosquitoes that had acquired virus by VNT. Twenty one percent (8 of 38) females receiving a BMnV prior to mating had DENV2 in their heads and 18.8% (6 of 32) of the non-BMnV female heads were positive (Fisher’s exact test P = 1.00). These data suggest that VNT-infections may disseminate to female salivary glands, but further work is required to show transmission.

## Discussion

Arboviruses are maintained by horizontal transmission (HT) between arthropod vectors and vertebrate hosts in nature. Arboviruses potentially can be transmitted vertically in the vector population from mother to offspring, which is a possible maintenance mechanism during adverse conditions for HT. Dry seasons in tropical areas, cold seasons in temperate regions, or insecticide spraying campaigns can drastically reduce vector density and thus opportunities for HT [47]. In addition, arbovirus infections in vertebrates usually result in long-lasting protective immunity so that high levels of herd immunity will minimize HT following epidemics. Several hypotheses have been suggested to explain the maintenance of arboviruses during inter-epidemic periods and include virus re-introduction and amplification of the virus in unknown host species (i.e., reservoirs) [53]. VT and VNT are alternate transmission mechanisms DENVs might use to maintain themselves in a vector population independent of feeding on viremic humans [29]. Although male mosquitoes are not hematophagous, they can acquire virus by VT from an infected female parent. In experimental studies, *Aedes* male mosquitoes infected by virus injection into the hemoceol transmitted virus to non-infected adult females during mating leading to infected F1 progeny [54, 55]. VNT of arboviruses by male mosquitoes has been demonstrated in mosquito vectors of bunyaviruses [54], alphaviruses [56, 57], flaviviruses [58] and rhabdoviruses [59].^.^ VNT has been observed in *Ae*. *albopictus* by intrathoracically injecting males with DENV1 prior to mating [11]. However, no previous studies have reported VNT by male progeny of *Ae*. *aegypti* females that were naturally infected (*per os*) with DENV. A relatively small number of stably infected females could maintain virus prevalence at a constant level if germarium infection occurred, assuming that any detrimental effects of the infection (e.g., longevity, fecundity, and development) are balanced by horizontal transmission [60, 61]. Stabilized infections with California encephalitis serogroup viruses (*Orthobunyavirus*: *Bunyaviridae*) have been demonstrated in several *Aedes* mosquito species [60, 61].

Our studies show that DENV2 infection of GDLS *Ae*. *aegypti* mosquitoes can persist by VT and VNT. The infection rates obtained with orally infected females indicate that GDLS mosquitoes are highly susceptible to DENV2. GDLS show an increased DENV2 titer when mosquitoes take a second BMnV.

This study used two techniques to show DENV2 VT in GDLS *Ae*.*aegypti*: 1) RT-N-PCR for amplification of viral RNA and 2) IFA detection of DENV2 antigen. Infectious DENV demonstrated by IFA in mosquito C6/36 cells exposed to mosquito tissue homogenates. The latter assay is considered the gold standard of evidence for VT. Although PCR-based assays are rapid and have higher sensitivity than virus isolation, they are prone to contamination or amplification of virus sequence incorporated into the mosquito genome. Consequently, resulting data should be interpreted cautiously and outcomes confirmed by virus isolation or IFA. In this report, we used RT-N-PCR assays to confirm IFA results for the presence DENV2.

Most previous studies demonstrated VT after intrathoracic injection of the virus [62, 63]. The observed VTR in the GDLS strain could be due to a higher efficiency of initially infecting GDLS females with artificial blood meals containing biologically relevant virus titers. In our study, we initially infected mosquitoes via a blood meal and DENV2 VT was analyzed after the second gonotrophic cycle (E2-7d, E2-10d, and E2-21d). E2-F1 mosquitoes were analyzed as a counter-weight to previous studies that showed little to no VT occurring after the first oviposition cycle. Lack of VT from E1 females to F1 progeny could be due to poor virus dissemination to reproductive tissues before the first egg batch is produced and laid, and/or decreased permeability of virus to nulliparous ovaries during oogenesis [48]. These findings were consistent with earlier reports for other flaviviruses (Zika virus (ZIKV), yellow fever virus (YFV), and West Nile virus (WNV), which showed infected female mosquitoes vertically transmit virus after the second oviposition cycle [31, 64, 65].

VTR were higher when infected females received a second BMnV at 10 or 21 dpi. However, a significant difference in VTR was observed even when infected females received a second BMnV at 7 dpi (E2-7d). These observations were similar to reports for La Crosse virus [66] and WNV [64] where VT occurred only during the second or later ovarian cycles. Previous VT studies conducted with YFV and DENV1 reported that the VT actually decreased with successive ovarian cycle of infected mosquitoes [17, 21]. In contrast, Diallo et al. reported that females vertically transmitted DENVs during second and third ovarian cycles, with the rates being highest in the third cycle [67]. As previously stated, VT is probably rare in the first ovarian cycle probably because eggs are oviposited 3–8 days post infection before the virus disseminates to the reproductive tract. Delayed oviposition by *Cx*. *pipiens* females of 11–14 dpi and 25 dpi resulted in VT of St. Louis encephalitis virus during the first ovarian cycle [68]. In support, we observed DENV2 in the ovaries, oviducts and oocytes of E1 females only at later times post-infection (17 dpi).

Rearing temperatures, passage level of virus, viral strain and mosquito strain/species have all been reported to influence the efficiency of VT of flaviviruses [63, 68–70]. In our study, we used DENV2 JAM1409, a viral strain that is not a natural match to the geographic origin of our mosquitoes and passaged in cell culture multiple times. Future studies of VT and VNT should explore closer geographic matches of low generation mosquito and viral strains.

Overall, *Aedes* mosquitoes display higher VTR than *Culex* mosquitoes. *Aedes* eggs are generally more resistant to desiccation than *Culex* eggs [71], which may confer a selective advantage to vertically transmitted viruses. In addition, *Aedes* mosquitoes had higher VTRs in arid climatic conditions compared to equatorial or warm temperate climatic conditions [50]. This supports the hypothesis that VT could be a maintenance mechanism when conditions are adverse for HT, in agreement with earlier observations [47, 72, 73]. It had been reported that eVTR measured in immature developmental stages were higher than in the corresponding adults [13, 69]. *Aedes aegypti* larvae vertically infected with YFV [17], Kunjin virus, and Japanese encephalitis virus [74] appeared to be slower in their development and vertically infected larvae may also suffer lower survival, hence leading to lower infection prevalence in adults [13].

Previous experimental infections under laboratory conditions revealed DENV VTR of 1–4% [21, 75, 76]. Detection of VT in nature is rare, due primarily to the low frequency of adult *Ae*. *aegypti* females infected with DENV. However, the occurrence of VT in nature has been documented by detection of DENVs in adult males [23, 26]. Recently others have reported VT of ZIKV in *Ae*. *aegypti* and *Ae*. *albopictus* [65]. They evaluated VT in larval pools of perorally infected *Ae*. *aegypti* and *Ae*. *albopictus* adult female mosquitoes or in adults mosquitoes intrathoracically injected with ZIKV [31]. Although VTR in those experimental studies were low, the efficiency may be sufficient to allow ZIKV (and other flaviviruses) to persist within the vector population in eggs during hot dry periods or cold weather, when adult vectors are absent or in low numbers.

A mathematical model that investigated parameters conditioning natural transmission and persistence of DENVs suggested that observed VTR in field settings are insufficient to maintain the virus in nature [4]. The modeling further suggested that the eVTR must be at least 20–30% to allow virus maintenance in nature in the absence of human-mediated amplification of DENVs. In our studies, MFIR from E2-10d mothers was 1:36 by IFA and RT-N-PCR, values substantially higher than reported for ZIKV (1:290;[31]), and YFV (1:596;[17]). The eVTR for E2-10d-F1 adult, infected mosquitos was 14–15% (Table 1), reflecting VT efficiencies that approach those suggested in the mathematical modeling study for DENV maintenance in nature [4]. Experimental studies of flavivirus VT in mosquitoes have variable results and indicate multiple factors can affect the frequency of VT and FIRs. These factors include mosquito species and geographic strain, mosquito age and mortality rate, virus genotype, persistence of virus during transstadial transitions, virus assay method, larval rearing temperature, interval between initial infection and second blood meal, and the ovarian cycle [31, 46, 75, 77]. These factors could certainly lower the eVTR of *Ae*.*aegypti* in nature and their contributions to eVTR in a given vector population should be further studied. Nevertheless *Ae*. *aegypti* biology and virus-vector interactions may enhance their eVTR. These positive factors include the mosquito’s acquisition of blood meals every few days and their demonstrated ability to go through multiple ovarian cycles. DENV maintenance in the vector population could be enhanced further by virus persistence in diapaused embryoes, DENV infection of oocytes and ovaries after the females acquire additional bloodmeals, and the relative efficiency of VNT. We hypothesize these attributes may counter the negative factors and allow the virus to persist in the vector population from one season to the next.

Overall, VT of DENVs in *Ae*. *aegypti* may be underestimated as a potential force driving the epidemiology of DENV infection. It is possible that infected males could maintain the virus at a low threshold without causing outbreaks in human populations, and initiate new infections by VT and VNT within the vector population [56]. We did not observe any significant differences in the efficiency of VNT between mosquitos fed (BMnV) and not fed prior to mating as previously reported in *Ae*. *albopictus* [11]. We did not observe any difference in DIR in heads of fed versus non-fed mosquitoes. In our study, evidence for VNT in *Ae*. *aegypti* was further supported by microscopy studies of DENV2 infected males by detecting viral antigen the testes, seminal vesicle and accessory glands [52].

Our results clearly demonstrate that infected GDLS female mosquitoes can vertically transmit DENV2 to their progeny and support our hypothesis that DENV VT efficiency largely depends on the interplay between gonotrophic cycle and viral infection dynamics. Offspring produced during the second or later gonotrophic cycles display higher VTR than offspring produced during the first gonotrophic cycle. Vertically infected males that have DENV in their reproductive tracts can sexually transmit DENV to non-infected virgin GDLS females during mating. This study demonstrates the potential for VNT of DENV by male *Ae*. *aegypti* and the first study to naturally infect adult male mosquitoes via VT and then show VNT of virus to virgin, uninfected females. Future studies are required to determine if the disseminated infection in females infected during mating leads to epidemiologically relevant transmission of DENV to progeny or to humans in nature. Importantly, *Ae*. *aegypti* infections with flaviviruses constitute a tractable experimental system to further understand the genetics, biology and epidemiological consequences of VT and VNT.
